# Combining Position Weight Matrices and Document-Term Matrix for Efficient Extraction of Associations of Methylated Genes and Diseases from Free Text

**DOI:** 10.1371/journal.pone.0077848

**Published:** 2013-10-16

**Authors:** Arwa Bin Raies, Hicham Mansour, Roberto Incitti, Vladimir B. Bajic

**Affiliations:** 1 Computational Bioscience Research Centre (CBRC), Computer, Electrical and Mathematical Sciences and Engineering Division (CEMSE), King Abdullah University of Science and Technology (KAUST), Thuwal, Saudi Arabia; 2 Bioscience Core Laboratories, King Abdullah University of Science and Technology (KAUST), Thuwal, Saudi Arabia; Beijing Institute of Genomics, Chinese Academy of Sciences, China

## Abstract

**Background:**

In a number of diseases, certain genes are reported to be strongly methylated and thus can serve as diagnostic markers in many cases. Scientific literature in digital form is an important source of information about methylated genes implicated in particular diseases. The large volume of the electronic text makes it difficult and impractical to search for this information manually.

**Methodology:**

We developed a novel text mining methodology based on a new concept of position weight matrices (PWMs) for text representation and feature generation. We applied PWMs in conjunction with the document-term matrix to extract with high accuracy associations between methylated genes and diseases from free text. The performance results are based on large manually-classified data. Additionally, we developed a web-tool, DEMGD, which automates extraction of these associations from free text. DEMGD presents the extracted associations in summary tables and full reports in addition to evidence tagging of text with respect to genes, diseases and methylation words. The methodology we developed in this study can be applied to similar association extraction problems from free text.

**Conclusion:**

The new methodology developed in this study allows for efficient identification of associations between concepts. Our method applied to methylated genes in different diseases is implemented as a Web-tool, DEMGD, which is freely available at http://www.cbrc.kaust.edu.sa/demgd/. The data is available for online browsing and download.

## Introduction

DNA methylation is one of the widely-studied [1-3] epigenetic modifications. Gene methylation can significantly affect the expression of genes by influencing their transcription [4]. Aberrant DNA methylation is found to be associated with cancer and in some cases with tumorigenesis, tumor stage, and antitumor treatment response [5]. DNA methylation is found to be an important utility to understand genetic mechanisms of tumorigenesis, and very useful for cancer diagnosis, cancer treatment or for prediction of anti-cancer treatment outcomes [5]. Besides cancer, DNA methylation is associated with many other diseases [6], for example, auto-immune diseases, neurodevelopmental disorders, and aging.

Associations between methylated genes and diseases have been investigated in several recent studies [7-9]. Moreover, a lot of information about methylated genes in specific diseases has been published during the last few decades. The need to disseminate this information motivated development of several DNA methylation databases, such as: DiseaseMeth [10], PubMeth [11], MethyCancer [12], MethDB [13,14], MethylomeDB [15], NGSmethDB [16], MeInfoText [17] and MeInfoText 2.0 [18]. There is only partial overlap of information between these different resources. These databases provide information on methylated genes associated with specific diseases, where this information is obtained by various methods. No publicly accessible tool exists that allows for the search for such information in free text submitted by users, which would enable researchers greater flexibility and acquiring information from the most recent and diverse literature.

In general, automated identification of useful information from free text is very attractive due to a large volume of existing textual information in digital format. Association between different concepts is a useful form of information and efficient extraction of such associations can benefit from text mining approaches that utilize the ordering of words in sentences. In order to extract such associations automatically from text, text must be represented in a structured format. The most common approach for structured text representation is the bag-of-words in which documents or sentences are represented as a list of words [19,20] by using a document-term matrix (DTM) [21]. The bag-of-words approach has been successfully applied for text classification, text clustering, and information retrieval [20]. This approach is based on the assumption that the position/ordering of words in a sentence is irrelevant [20]. Such assumption is largely unrealistic because the order of words in a sentence may convey different messages but any two sentences that include the same words in different order are indistinguishable using this approach. However, due to its simplicity, the bag-of-words approach is widely used and is considered computationally efficient [22]. Current text mining studies still rely on the bag-of-words approach, although it ignores the word order information [19]. Some fields such as text compression, named entity recognition, association extraction, and generally natural language processing may require preserving the original order of words in text [19,22] for increased recognition accuracy. 

Here we introduce a new methodology for text representation and feature generation based on position weigh matrices (PWMs), a concept that is widely used in sequence analysis [23]. To apply PWMs in text mining, we segment the sentences based on the concepts and relationship terms that are used as delimiters to distinguish between different segments in sentences. We used PWMs to capture the frequency of words in each segment in sentences, and then used PWMs to compute matching scores for other sentences. The methodology we developed is generic in nature and can be applied to several types of association extraction problems from free text. In this study, we provide detailed explanation on applying PWMs for text representation and feature generation for a specific problem of extracting associations between methylated genes and diseases from free text.

We compared the performance between the DTM and the PWMs approaches applied in combination with several machine learning algorithms where the performance is evaluated using manually-classified datasets. Then we evaluated the performance when the two approaches are applied together. The best achieved results are based on the random forest machine learning algorithm and a combination of DTM with PWMs. Using 10-fold cross-validation on a manually-classified dataset, which consists of 1124 abstracts, 2361 positive and 2302 negative sentence patterns, our method achieved F-score and accuracy of over 84% and over 83%, respectively. Using a completely separate manually-classified testing set, which included 72 abstracts, 100 positive and 100 negative sentence patterns, the F-score and accuracy of over 88% and over 87%, respectively, were obtained.

The method we developed is implemented in the context of extracting methylated genes in diseases as a Web-tool, Dragon Extractor of Methylated Genes in Diseases (DEMGD), which is free for academic and non-profit users at http://www.cbrc.kaust.edu.sa/demgd/. DEMGD offers a user-friendly interface for extraction of associations of methylated genes and diseases and text annotation with respect to genes, diseases and methylation words from text submitted by users. This tool facilitates discovery of these associations from any free text and aims to support research in this domain. Also, in this study, we contribute to the text-mining community a large dataset (1196 abstracts) of manually-curated genes, diseases and methylation words mentions. The dataset is available at http://www.cbrc.kaust.edu.sa/demgd/ for online browsing and download.

## Methods

### Problem formulation

In our system, we consider three categories of words related to genes, diseases, and methylation. We refer to these three categories as ‘concepts’. In order to extract the associations between methylated genes and diseases, we only consider the cases where the three concepts appear in the same sentence. We call the order in which the three concepts appear in sentences a ‘pattern order’. Table 1 shows all possible (six) different pattern orders. We call an instance of a pattern order a 'pattern'. Below we provide an example of a sentence from a PubMed (http://www.ncbi.nlm.nih.gov/pubmed) abstract (PubMed: 21693594) that includes a pattern (marked as italic and underlined):

**Table 1:** Illustration of pattern order.

	...	First Concept	...	Second Concept	...	Third Concept	...
1	...	<Disease>	...	<Gene>	...	<Methylation Word>	...
2	...	<Disease>	...	<Methylation Word>	...	<Gene>	...
3	...	<Gene>	...	<Disease>	...	<Methylation Word>	...
4	...	<Gene>	...	<Methylation Word>	...	<Disease>	...
5	...	<Methylation Word>	...	<Disease>	...	<Gene>	...
6	...	<Methylation Word>	...	<Gene>	...	<Disease>	...

The table shows the six different patterns orders that can appear in sentences. For example, the first patterns order means that the disease is mentioned first in the sentence, the gene is mentioned second and the methylation word is mentioned last.

The CPG island in the *FILIP1L* <Gene> promoter was heavily *methylated* <Methylation Word> in *ovarian cancer* <Disease> cells. 

In this example, the pattern is (FILIP1L, methylated, ovarian cancer), while the pattern order is (<Gene>, <Methylation word>, <Disease>). 

We represent the association extraction task as a binary classification problem. Patterns that express associations between methylated genes and diseases are named ‘positive patterns’, while those that do not express such associations are named ‘negative patterns’. It is possible that a sentence contains more than one pattern, and a sentence may contain both negative and positive patterns as well. Below is an example of a sentence from a PubMed abstract that contains negative and positive patterns:

We found that *methylation* <Methylation Word> of the *CRY1* <Gene> promoter was detectable in *Parkinson's* *disease* <Disease>, but absent in *PER1* <Gene> promoter.

The pattern (methylation, CRY1, Parkinson's disease) appears as a positive pattern in the above sentence, while the pattern (methylation, Parkinson's dis-ease, PER1) is a negative pattern. Accordingly, we developed a methodology to identify negative and positive patterns even if they appear in the same sentence.

### Text pre-processing

**Figure 1:**
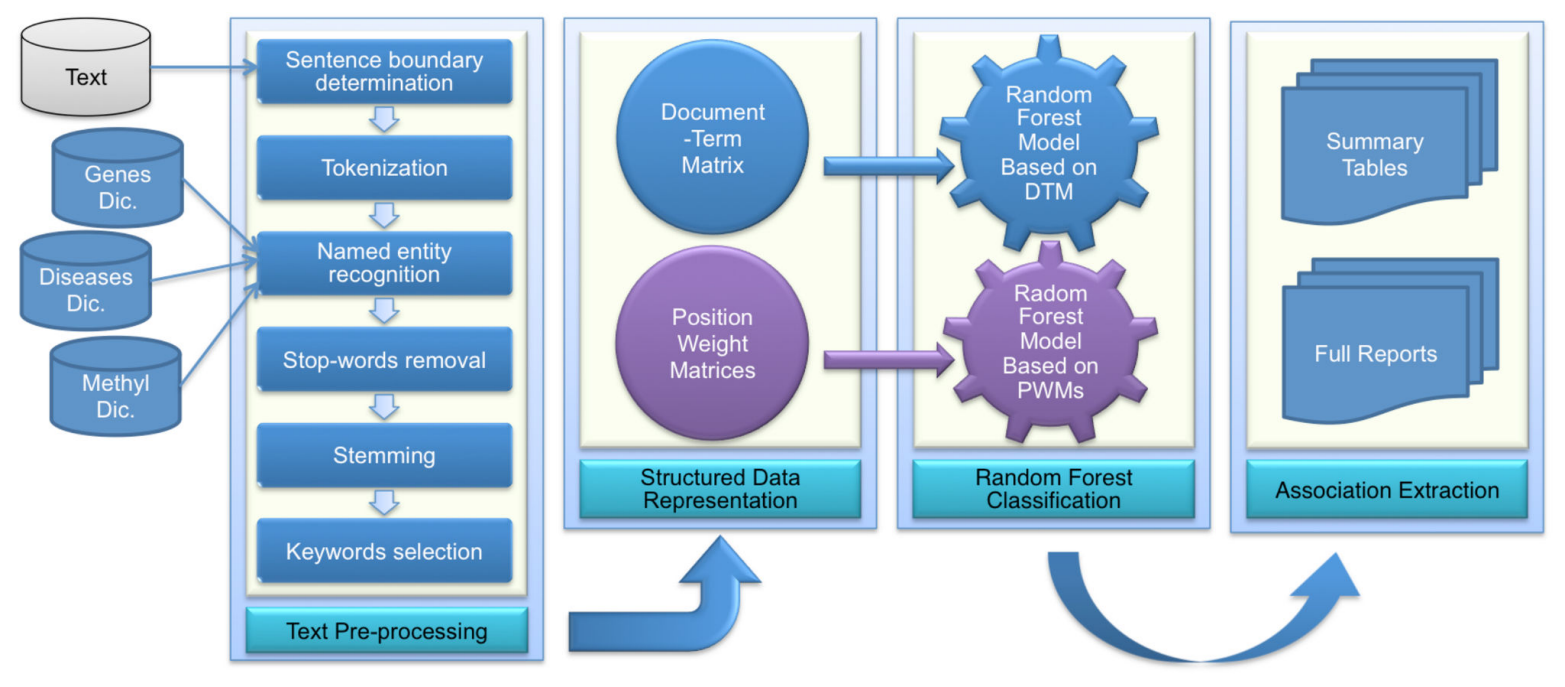
DEMGD system architecture. The input to the system is the Input Text, and the output is Summary Tables and Full Reports. The system consists of four modules: Text Pre-processing, Structured Data Representation, Classification and Associations Extraction.

Figure 1 depicts the structure of DEMGD. The text pre-processing module of DEMGD implements six steps. Firstly, sentence boundary determination is performed by the set of rules from Weiss et al. [24] to determine the end of sentences. The second step is tokenization that breaks sentences into words, and we used the following rules to determine the Boundary Of Words (BOW):

 Newline, tab, space, !, and ? are always BOW

• Period followed by whitespace is BOW
• If the word to which “, ‘, (, ), [, ], <, or >, is attached at the end or the beginning does not include the matching punctuation in the middle of the word, it is BOW (for example, the parenthesis are considered part of the name of P14(ARF) gene, so the parenthesis in this case cannot be used as a boundary of a word)
• Otherwise, it is not BOW.

The third step is named entity recognition that aims to identify genes, diseases and methylation words. We used three manually-compiled dictionaries (for genes, diseases and methylation words). These dictionaries include all various ways an entity can be expressed. For example, the diseases dictionary includes ‘Type 1 Diabetes’, ‘Diabetes Type-1’, ‘Diabetes Type 1’, ‘Diabetes Type1’, ‘Type1 Diabetes’ and ‘Type-1 Diabetes’. This is done in order to maximize the recall rate of named entity recognition step. We used dictionary-based longest matching technique to extract multi-word entities (e.g., the disease ‘Diabetes Type 1’ consists of three words). One of the main requirements of a NER system is determining the boundary of multi-word entities. If a sentence includes ‘Diabetes Type 1’, it is inaccurate to extract only ‘Diabetes’ instead of the whole entity ‘Diabetes Type 1’. Therefore, the following steps describe the logic of dictionary-based longest matching technique:

1. Take the first word in the sentence ‘w_1_’.
2. Check if ‘w_1_’ exists in the diseases dictionary.
3. If w_1_ exists in the dictionary, then take the following word ‘w_2_’, and check if the sequence of words ‘w_1_ w_2_’ exists in the dictionary.
4. Repeat step 3 until we add a word ‘w_n_’ such that the sequence of words ‘w_1_ w_2_ … w_n-1_ w_n_’ does not exist in the dictionary.
5. The sequence ‘w_1_ w_2_ … w_n-1_’ is determined to be a named entity.
6. Repeat the steps 1 to 5 using the genes and the methylation words dictionary.
7. Repeat the steps 1 to 6 starting from word ‘w_n_’.

We evaluated the performance of NER step on our test set T used in the original manuscript. The precision/recall/F-score are 94.48/90.13/92.26, 98.61/100.00/99.30 and 84.69/69.17/76.15% for genes, methylation words and diseases, respectively.

The forth step is stop-words elimination to remove common words that may not contribute to discrimination between classes, and this step is performed using a stop-words list. The fifth step is stemming in which all suffixes and prefixes are removed from words, and this step is performed using Porter Stemmer [25] (MATLAB version) (http://tartarus.org/martin/PorterStemmer/). The last step is keywords selection. Keywords are determined by using information gain that estimates the information gained when predicting a class based on the presence (or absence) of a specific word in a sentence. The information gain is determined as in [26] (see Information S1).

### Structured data representation

This DEMGD module converts free, unstructured text into a structured representation. DEMGD implements two types of representations where each representation provides a different aspect of statistical information. The first type implements DTM representation, whereas the second type implements PWM representation. The following two subsections explain implementation of DTM and PWM approaches separately. Then Hybrid Approach subsection, explains how the two approaches are combined in DEMGD.

### DTM

In DTM, each column corresponds to a keyword, and rows correspond to sentences. The elements are represented using (i) binary, (ii) frequency, and (iii) TF-IDF values. The first mechanism is based on using binary values where 1 represents words that appear in sentences, and 0 represents words that do not appear in sentences. The second mechanism uses frequency values that represent the number of times a word appears in a sentence. The last mechanism uses term-frequency inverse-document-frequency (TF-IDF) with z-score normalization. TF-IDF is defined as in [27], and Z-score normalization is defined as in [28] (see Information S1). These are used in connection with different machine learning algorithms. We used frequency values representation of DTM described previously for Naïve Bayes, binary representation of DTM for rule generation algorithms, and TF-IDF with z-score normalization for decision trees, KNN, and SVM (see Classification Module subsection).

### PWMs

So far, PWMs [29] have been used to solve different problems in bioinformatics such as motif discovery [30], binding site identification [31], etc. Here we introduce a novel application of PWMs for text mining.

**Figure 2:**
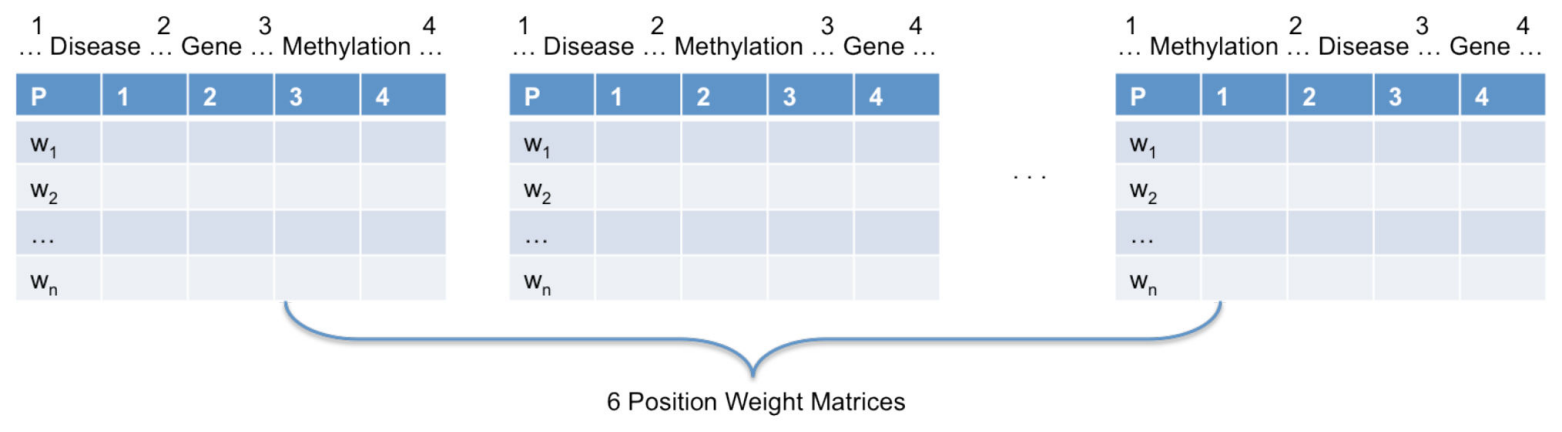
The structure of PWMs. We can generate six PWMs, and each matrix corresponds to a pattern order. For example, the first PWM to the left corresponds to the pattern order (<Disease>, <Gene>, <Methylation Word>). Each row corresponds to a word, and each column corresponds to a segment, and cells of the matrix represent the frequency of words in each segment.

The fundamental idea underlying this approach is to align sentences along several key concepts so as to segment sentences to different parts. In our case we consider the three concepts: genes, diseases and methylation words. Because there are six different pattern orders of these three concepts (as illustrated in Table 1), we generated six different PWMs, and each matrix represents a specific pattern order (see Figure 2). Each row in a PWM represents a specific word. The number of columns of each PWM is four, because for each pattern order, we can distinguish four segments in each sentence. For example, let us assume that the pattern order is <gene> <methylation word> <disease>. We will consider four segments (<segment 1>, <segment 2>, <segment 3>, <segment 4>) of the sentence to generate a PWM for this pattern order:

<Segment 1> <gene> <segment 2> <methylation word> <segment 3> <disease> <segment 4>

The first column of a PWM represents the frequency of words in the first segment. Similarly, the second, third and fourth columns represent the frequency of words in the second, third and fourth segments, respectively. We generated 12 different PWMs: six PWMs for the negative class (called ‘negative PWMs’), and six PWMs for the positive class (called ‘positive PWMs’). 

#### Dictionary Generation

Before generating the PWMs, we need to generate two dictionaries for each class of sentences. The positive dictionary includes words that appear frequently in the positive sentences, whereas the negative dictionary includes words that appear frequently in the negative sentences. The following steps are performed to determine if a word belongs to the positive dictionary or the negative dictionary:

1. Compute the frequency of the word in the positive class (F_pos_) by dividing the number of positive sentences that contain the word by the total number of positive sentences.
2. Compute the frequency of the word in the negative class (F_neg_) by dividing the number of negative sentences that contain the word by the total number of negative sentences.
3. If F_pos_ > F_neg_, the word belongs to the positive dictionary; otherwise, the word belongs to the negative dictionary. If F_pos_ = F_neg_, the word belongs to both dictionaries.

The number of rows in the positive and negative PWMs is determined by the number of words in the positive and negative dictionaries, respectively. 

#### PWMs Generation from text

To generate PWMs from a collection of positive sentences or negative sentences, we first identify the pattern that appears in the sentence. Then we identify words in each segment. After that, we update the PWM that corresponds to the corresponding pattern order. In the following example, the first segment is before the gene concept, which includes the words (‘CPG’ and ‘island’):

The CPG island in the *FILIP1L* <Gene> promoter was heavily *methylated* <Methylation Word> in *ovarian* *cancer* <Disease> cells (PubMed: 21693594).

**Figure 3:**
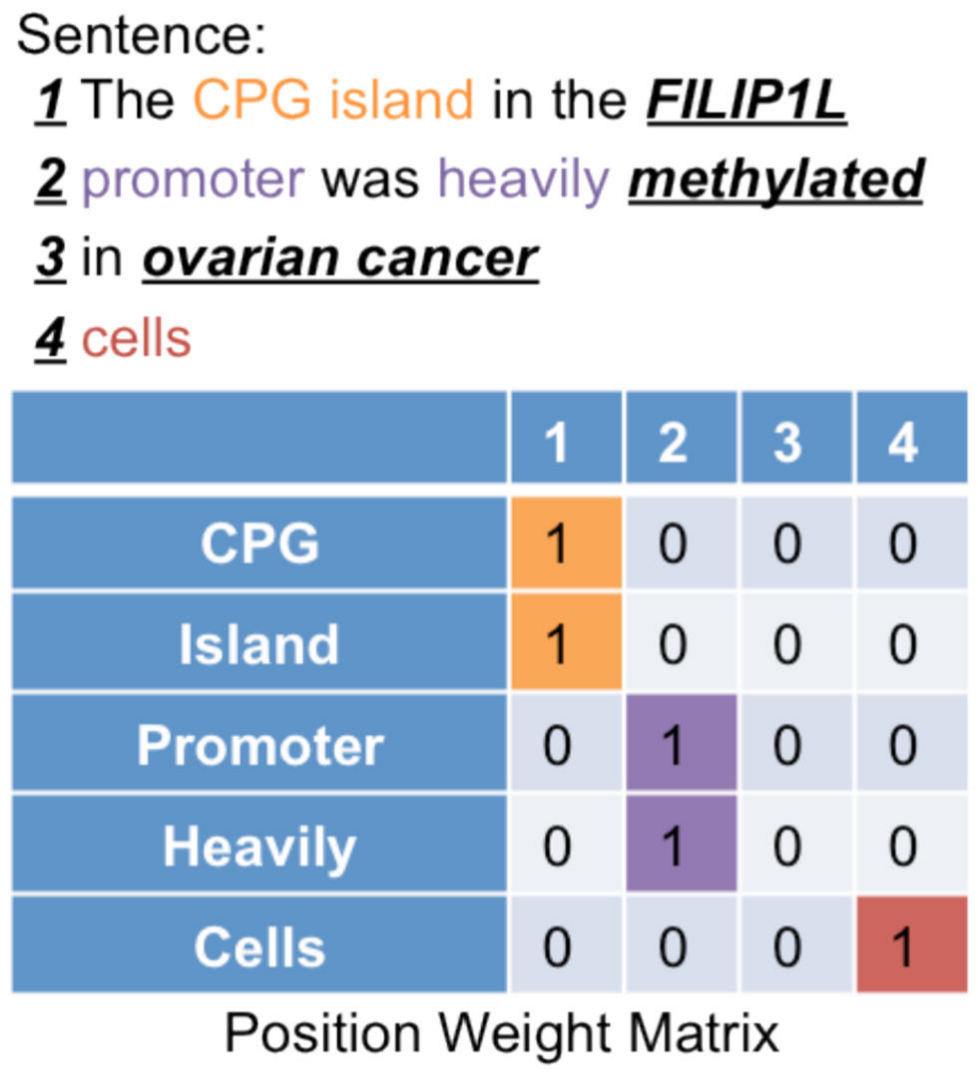
PWM generation. The PWM summarizes frequency of words in each segment. For example, the words ‘CPG’ and ‘island’ appear in the first segment of the sentence, so the rows that correspond to these words and the first column is incremented by one. Similarly, the same step is applied to words in the remaining three segments. The same matrix is updated using other sentences with the same pattern order.

So the matrix elements intersecting with the rows that correspond to ‘CPG’ and ‘island’, and the first column will be incremented by one. Similarly, the second, third and fourth columns are updated. Figure 3 shows how the PWM, which represents this specific pattern order, is updated. The next step after generating the PWMs is to normalize the matrices by dividing frequency in each cell in a column by the total sum along the column. 

#### Computing matching scores for sentences

The main application of PWMs is to match sentences that contain a specific pattern order with the corresponding PWMs to compute the matching scores for the pattern in the sentence. We use the following example to explain how to compute these scores. Consider a sentence that contains one pattern (MIR203, methylated, MM) in two pattern orders ((<Gene>, <Methylation Word>, <Disease>) and (<Gene>, <Disease>, <Methylation Word>)):

Promoter of *MIR203* <Gene> was found *methylated* <Methylation Word> in approximately 25% *multiple* *myeloma* <Disease> cell lines but not *methylated* <Methylation Word> in normal controls

**Figure 4:**
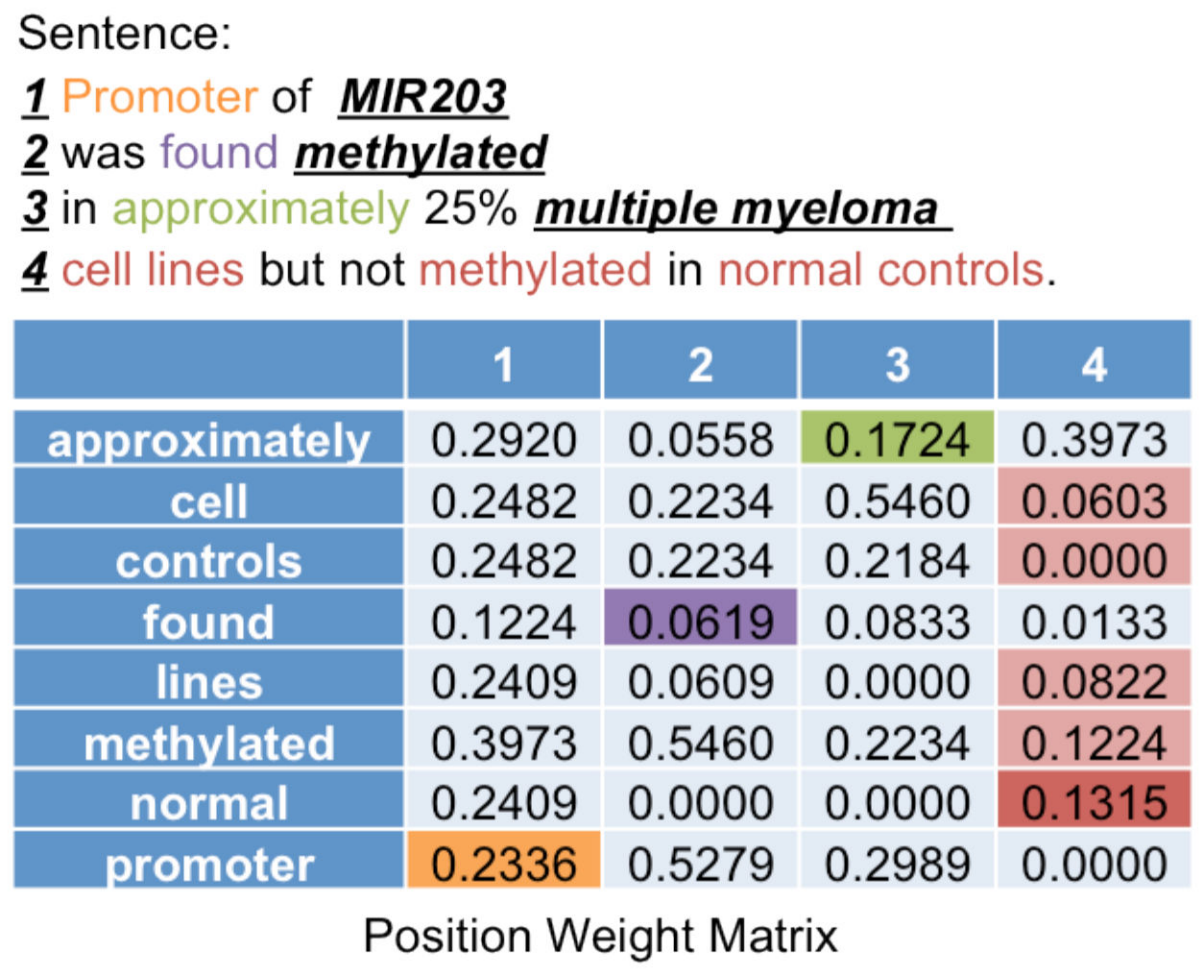
Computing the scores. The figure shows an example of a normalized PWM. To compute the score, we sum the weights of one word from each column. For example, the word ‘promoter’ appears in the first segment, so we take its weight from the first column in the PWM. The same step is applied to the second, and the third segments. However, five words appear in the last segment, so we take maximum weight. The score of the pattern is 0.2336+0.1619+0.1724+0.1315=0.5994.

The pattern in the sentence in this example will be given four scores. Two scores will be given using the positive and negative PWMs that correspond to the first pattern order (<Gene>, <Methylation Word>, <Disease>), and two scores will be given using the positive and negative PWMs that correspond to the second pattern order (<Gene>, <Disease>, <Methylation Word>). As an example, Figure 4 shows how to compute the score for the pattern in the sentence for this example using a positive PWM that corresponds to the first pattern order (<Gene>, <Methylation Word>, <Disease>). To compute the scores for the sentence in this example, the following steps are performed:

1. Identify the pattern that appears in the sentence (MIR203, methylated, MM). These concepts partition the sentence to segments.
2. Identify words in each segment. The words ‘promoter’, ‘found’ and ‘approximately’ appear in the first, second and third segments, respectively, and the words ‘cell’, ‘controls’, ‘lines’, ‘methylated’ and ‘normal’ appear in the fourth segment.
3. Determine the weight of each word from each segment. In the first, second and third segments, we find (‘promoter’, 0.2336), (‘found’, 0.619), and (‘approximately’, 0.1724), respectively.
4. If there are several words in one segment, we chose the maximum weight in the segment. In the fourth segment, we find (‘cell’, 0.0603), (‘controls’,0), (‘lines’,0.0822), (‘methylated’, 0.1224) and (‘normal’, 0.1315), so the maximum weight is 0.1315 for the word ‘normal’ and this one is chosen.
5. Sum the four weights of the four segments to get the final score of the pattern in the sentence. For this example we get 0.2336 + 0.619 + 0.1724 + 0.1315 = 1.1565.

We should note that there are different ways to compute the score in case of several words appearing in the same segment, such as summing all weights or multiplying the respective probabilities of occurrence of the words, etc.. However, the method we described above is significantly different from those customary for sequence analysis and it achieved on our data the best results, so we implemented it for the system. The analogous steps are followed to compute the score for the sentence using the negative PWM. 

#### Examples of different cases for scoring

In general, we can get up to twelve scores for each pattern in a sentence. For example, the following sentence contains one pattern (BRCA1, methylation, breast cancer) in six pattern orders:

By examining *BRCA1* <Gene> *methylation* <Methylation Word> in *breast* *cancer* <Disease> patients, we found heavy *methylation* <Methylation Word> in the promoter of *BRCA1* <Gene>, which indicates the associations between *BRCA1* <Gene> *methylation* <Methylation Word> and *breast* *cancer* <Disease> prognosis. 

The pattern in the previous example will be given twelve scores. Two scores will be given using the positive and negative PWMs that correspond to the first pattern order (<Gene>, <Methylation Word>, <Disease>). Similarly, the remaining ten scores will be given using the five positive PWMs and five negative PWMs that correspond to the remaining five pattern orders.

If a sentence contains a pattern that appears several times in the same pattern order, the scores are summed. For example, in the following sentence, the pattern (BRCA1, methylation, breast cancer) appears two times in one pattern order (<Disease>, <Gene>, <Methylation Word>):

By examining *breast* *cancer* <Disease> patients, *breast* *cancer* <Disease> prognosis is found to be associated with *BRCA1* <Gene> promoter *methylation* <Methylation Word>

The pattern (BRCA1, methylation, breast cancer) will be given a score by considering

By examining *breast* *cancer* <Disease> patients, breast cancer prognosis is found to be associated with *BRCA1* <Gene> promoter *methylation* <Methylation Word>

#### And another score by considering

By examining breast cancer patients, *breast* *cancer* <Disease> prognosis is found to be associated with *BRCA1* <Gene> promoter *methylation* <Methylation Word>

Since these scores are generated with respect to the same pattern order, the scores will be summed. 

If a sentence contains several genes and/or diseases, the sentence is duplicated several times with respect to each pattern, and the scores will be computed for each pattern separately. For example, the following sentence contains two patterns (BRCA1, methylated, ovarian cancer) and (FILIP1L, methylated, ovarian cancer):

*BRCA1* <Gene> and *FILIP1L* <Gene> are found to be *methylated* <Methylation Word> in *ovarian* *cancer* <Disease>.

The scores will be computed for each pattern separately by duplicating the sentence two times to represent only one pattern as follows

*BRCA1* <Gene> and FILIP1L are found to be *methylated* <Methylation Word> in *ovarian* *cancer* <Disease>.

BRCA1 and *FILIP1L* <Gene> are found to be *methylated* <Methylation Word> in *ovarian* *cancer* <Disease>.

### Pattern representation

**Figure 5:**
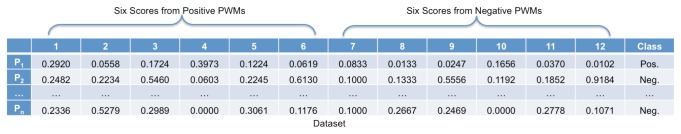
Dataset representation using PWMs. Each pattern in a sentence is represented with twelve features and a class label. The first six features correspond to the scores generated from the positive PWMs, and the following six features correspond to the scores generated from the negative PWMs.

We represent each pattern in a sentence with twelve features based on the scores and a class. The twelve features consist of six scores from the six positive PWMs and six scores from the six negative PWMs. Figure 5 shows the features used to represent the dataset based on the generated scores. 

The main advantage of PWMs approach is its ability to score patterns in a sentence independently from each other so that each pattern may get different scores. For example, there are positive and negative patterns in the following sentence:

We found that *methylation* <Methylation Word> of the *CRY1* <Gene> promoter was detectable in *Parkinson's* *disease* <Disease>, but absent in *PER1* <Gene> promoter.

The pattern P1 (methylation, CRY1, Parkinson's disease) is a positive pattern, but the pattern P2 (methylation, Parkinson's disease, PER1) is a negative pattern. Table 2 shows the features of the sentence with respect to each pattern. This way the features of the patterns have different values even though the patterns appear in the same sentence. This approach allows machine learning algorithms to distinguish between positive and negative patterns even if they appear in the same sentence. Interestingly, DTM approach does not distinguish between positive and negative patterns within the same sentence.

**Table 2:** Features of two patterns in the same sentence.

	1	2	3	4	5	6	7	8	9	10	11	12	Class
P1	0	0	0.069	0.0219	0	0	0	0	0.0123	0.0331	0	0	Pos.
P2	0.0511	0	0.069	0.0219	0.0102	0	0.0167	0	0.0123	0.0331	0.0185	0	Neg.

The two patterns P1 and P2 appear in the same sentence, but the first one is positive and the second one is negative. The features generated by the PWMs can represent the two patterns differently even though they appear in the same sentence. Pos: Positive; Neg: Negative

### Hybrid approach

This approach combines the previous two approaches (DTM and PWM). The hybrid approach requires developing two classification models. The first classification model is generated by applying the DTM approach to sentences with one pattern only, and the second classification model is generated by applying the PWM approach to sentences with several patterns. For each sentence in the testing set we first determined the number of patterns it contains. Sentences that do not contain any pattern are discarded. The first model trained using features obtained via DTM approach is applied if a sentence has only one pattern. The second model trained using features obtained via PWM approach is applied if the sentence contains several patterns.

### Classification module

Using the structured representation of patterns in each sentence, patterns will be classified as either positive or negative by the Classification Module. We compared the performance of several different classifiers on our data in the search for the most efficient machine learning model including rule-based ones (FOIL [32], CPAR [33], CMAR [34], CBA [35], PRM [33], and TFPC [36]), K-nearest neighbor (KNN) [37], support vector machines (SVM) [38], decision trees (C4.5 [39], random forest [40], and random tree [41]) and Naïve Bayes [42]. We used WEKA [43] implementation (http://www.cs.waikato.ac.nz/ml/weka/downloading.html) of Naïve Bayes and decision trees, LUCS-KDD implementation of rule generation algorithms (http://cgi.csc.liv.ac.uk/~frans/KDD/Software/), LIBSVM [44] for SVM implementation (MATLAB version) (http://www.csie.ntu.edu.tw/~cjlin/libsvm/#matlab), and MATLAB (2011b version) implementation of KNN. We implemented the classification module based on the classification algorithm that performed the best in comparison, which in our case appears to be the random forest models.

### Association extraction module

Finally, the patterns that were predicted by the classification model to be positive are considered to represent potential associations. The Association Extraction Module organizes the associations into a summary table and a full report. The summary table lists for each sentence: a/ genes, diseases and methylation words that appear in the sentence, b/ the corresponding sentence, and c/ the abstract PMID where the sentence appears (in case PubMed abstracts were submitted to the systems). The full report lists for each full article or abstract: a/ genes, diseases and methylation words that appear in the sentence, b/ the corresponding color-tagged sentence, along with colored tagging of all genes, diseases and methylation words that appear in a full article or abstract.

### Data acquisition

The approaches discussed here required an initial creation of a dataset for developing and testing machine learning association identification models. 1,124 abstracts were extracted from PubMed database, from which we extracted 2,049 sentences where each contained at least one pattern. Because some sentences contain more than one pattern, a total of 4,663 different patterns were obtained from these sentences. 

Through hand-curation, we classified the patterns into negative or positive. 49% of patterns were negative (2302 negative patterns), while the remaining 51% of patterns were positive (2361 positive patterns). We used 30% of the sentences to generate PWMs, and we call that set of sentences set P. The remaining 70% of sentences we call set C and it is used for 10-fold cross-validation of machine learning algorithms. In addition, we generated another set, set T, which contains 75 sentences extracted from 42 abstracts (separate from 1,124 abstracts described previously). These produced 200 manually-classified patterns (100 negative and 100 positive).

### Classification performance measures

For performance evaluation, we computed the following performance measures presented in Table 3. There, TP (true positive) indicates that a positive pattern is predicted as positive, while FN (false negative) indicates that the positive pattern was predicted as negative. On the other hand, TN (true negative) indicates that a negative pattern is predicted as negative, while FP (false positive) indicates that a negative pattern was predicted as positive. TP, FP, TN and FN were calculated for the cases when entities are identified.

**Table 3:** Classification performance measures.

Measure	Equation
Accuracy	(TP+FP) / (TP+FP+TN+FN)
F-score	(2*Precision*Recall) / (Precision + Recall)
Precision	TP / (TP+FP)
Recall	TP / (TP+FN)
Specificity	TN / (TN+FP)

The table shows the performance measures. TP: True Positive; FP: False Positive; TN: True Negative; FN: False Negative.

## Results

In this section, we compared the performance of several machine learning models using DTM, PWMs and hybrid approaches.

### Classification performance using the PWMs approach

In this approach we used the features generated by PWMs to train machine learning models. The models must classify each pattern as positive or negative even if multiple patterns appear in the same sentence. PWMs were generated from set P. 10-fold cross-validation on set C is used to evaluate all algorithms. We tested range of parameters for each algorithm (Table S1 in Supporting Information) and recorded the best performance with the corresponding parameters. We began by evaluating the performance of algorithms when applied on sentences with multiple patterns from set C. The best accuracy (details about used performance measures are available in Table S1 in Supporting Information) achieved was 85.5% by a random forest model. However, when we applied the algorithms on sentences from set C that contain only one pattern, we noticed a decrease in performance. The best accuracy achieved was 69.2%, again by a random forest model. Then we applied the algorithms on the entire set C that contains sentences with one pattern and sentences with multiple patterns. The random forest model outperformed the other algorithms and achieved 81.5% accuracy. Table 4 summarizes the best performance of all algorithms using PWMs approach. 

**Table 4:** Performance of PWM approach after 10-fold cross-validation of algorithms using sentences with multiple patterns from set C, sentences with single patterns only form set C and entire set C after the named entity recognition.

	Algorithm	Accuracy	Precision	Recall	Specificity	Parameters
sentences with multiple patterns from set C	Random forest	85.5%	85.8%	85.0%	86.0%	10 decision trees and 12 random features
	SVM	69.6%	69.9%	69.1%	70.1%	Polynomial kernel, cost = 8, gamma=0.5, coeff=8, degree=4
	KNN	70.1%	67.9%	75.0%	65.9%	Euclidean distance and 3 nearest neighbours
	C4.5	80.5%	79.4%	82.2%	78.8%	Confidence = 0.7
	Random Tree	85.2%	85.0%	85.3%	85%	12 random features selected
	Algorithm	Accuracy	Precision	Recall	Specificity	Parameters
sentences with one pattern only from set C	Random forest	69.2%	74.2%	79.1%	51.7%	15 decision trees and 4 random features
	SVM	70.3%	75.3%	80.2%	51.6%	Polynomial kernel, cost = 8, gamma=0.25, coeff=8, degree=4
	KNN	68.7%	71.5%	85.2%	49.7%	City block distance and 5 nearest neighbours
	C4.5	70.4%	71.4%	89.3%	37.4%	Confidence = 0.1
	Random Tree	66.8%	73.4%	74.9%	52.6%	4 random features selected
	Algorithm	Accuracy	Precision	Recall	Specificity	Parameters
Sentences from entire set C	Random forest	69.2%	74.2%	79.1%	51.7%	15 decision trees and 4 random features
	SVM	70.3%	75.3%	80.2%	51.6%	Polynomial kernel, cost = 8, gamma=0.25, coeff=8, degree=4
	KNN	68.7%	71.5%	85.2%	49.7%	City block distance and 5 nearest neighbours
	C4.5	70.4%	71.4%	89.3%	37.4%	Confidence = 0.1
	Random Tree	66.8%	73.4%	74.9%	52.6%	4 random features selected

### Classification performance using the DTM approach

Here we used the features generated by DTM approach to train machine learning models. Considering that DTM approach cannot be used to classify several patterns if they are present in the same sentence, we applied DTM to classify sentences instead of patterns. If a sentence includes only one pattern, we label the sentence as negative if the pattern is negative; otherwise, the sentence is labeled as positive if the pattern is positive. However, if a sentence has several patterns, the sentence is labeled positive in case there is at least one positive pattern; otherwise, the sentence is labeled as negative (all patterns are negative). When we applied 10-fold cross-validation using all algorithms to the entire set C, a random forest model achieved the best performance with 80% accuracy. We noticed a slight decrease in performance when the algorithms were applied on sentences from set C with one pattern only. The best accuracy achieved was 77% by a random forest model. Table 5 summarizes the best performance of all algorithms using DTM approach. 

**Table 5:** Performance of DTM approach after 10-fold cross-validation of algorithms using entire set C and sentences with single patterns only form set C after the named entity recognition.

	Algorithms	Accuracy	Parameters
Entire set C	FOIL	71%	Gain = 80%
	CPAR	63%	Gain = 90%
	PRM	62%	Gain = 90%
	CMAR	68%	Support = 4% and confidence = 50%
	CBA	79%	Support = 0.25% and confidence = 90%
	TFPC	76%	Support = 1% and confidence = 40%
	Random forest	80%	No. of trees = 15, no. of random features = 10 and no. of keywords = 512
	C4.5	76%	Confidence = 0.1 and no. of keywords = 512
	Random Tree	74%	No. of trees = 1, no. of random features = 20 and no. of keywords = 512
	SVM	77%	Linear kernel, cost = 1, and no. of keywords = 256
	KNN	73%	City block distance, no. of neighbours = 1 and no. of keywords = 256
	Naïve Bayes	77%	No. of keywords = 256
	Algorithms	Accuracy	Parameters
Sentences with single patterns only	FOIL	53%	Gain = 60%
	CPAR	43%	Gain = 70%
	PRM	50%	Gain = 70%
	CMAR	32%	Support = 0.25% and confidence = 40%
	CBA	65%	Support = 2% and confidence = 80%
	TFPC	65%	Support = 4% and confidence = 70%
	Random forest	77%	No. of trees = 15, no. of random features = 15 and no. of keywords = 512
	C4.5	74%	Confidence = 0.1 and no. of keywords = 128
	Random Tree	74%	No. of trees = 1, no. of random features = 26 and no. of keywords = 512
	SVM	74%	Radial kernel, cost = 1, gamma = 0.0078, and no. of keywords = 128
	KNN	71%	Cosine distance, no. of neighbours = 1 and no. of keywords = 128
	Naïve Bayes	75%	No. of keywords = 512

### Classification performance using the hybrid approach

Due to the fact that PWM approach worked best for sentences with multiple patterns, while DTM worked best for sentences with single patterns, we implemented a hybrid approach in which we trained two classification models. We evaluated the performance of five different algorithms. Table 6 shows the accuracy of several machine learning algorithms after applying the hybrid approach with 10-fold cross-validation on set C. The best performance achieved was with the random forest models and the achieved performance of the hybrid approach was 83.5% and 84.7% for accuracy and F-score, respectively.

**Table 6:** 10-fold cross-validation accuracy of algorithms after applying them using the hybrid approach on entire set C after the named entity recognition.

Algorithms	Recall	F-score	Accuracy	Precision	Specificity
Random Forest	86.27%	84.77%	83.5%	83.33%	80.35%
C4.5	80.35%	79.82%	78.38%	79.31%	76.13%
Random Tree	83.95%	83.57%	82.64%	83.57%	81.22%
SVM	70.37%	68.85%	67.41%	67.4%	64.45%
KNN	75.84%	73.05%	70.31%	70.46%	64.07%

### Classification performance on an independent testing set

From the previous analysis, we determined that the random forest models with the hybrid approach achieved the best performance in 10-fold cross-validation. A separate testing set T is used to evaluate performance of the hybrid method (see Table 7). 

**Table 7:** Performance of the hybrid approach using a separate testing set T after the named entity recognition.

Algorithms	Recall	F-score	Accuracy	Precision	Specificity
Hybrid approach with Random Forest	99.00%	88.79%	87.50%	80.49%	76.00%

### Comparison with other systems

There are no publicly available tools that allow for extraction of methylated genes in different diseases, based on submitted text, thus it is not possible to make the comparison of our results to such methods. Other computational methodologies for extracting associations between methylated genes and specifically cancer [17,18] have been used in compiling MeInfoText [17] and its successor, MeInfoText 2.0 [18] databases. The difference between these systems and DEMGD is that these systems allow users to retrieve associations between methylated genes and diseases from databases, but our system allows users to submit text (abstracts or full articles) and extract associations between methylated genes and diseases from the submitted text. MeInfoText databases provide users with information about the associations of methylated genes and different cancers. For the text mining system used to compile MeInfoText, the performance has been evaluated using 75 associations, and the reported precision and recall are 99% and 93%, respectively. We note that conclusions about the performance derived using only 75 associations is rather inaccurate and not comparable to performance of our method assessed on significantly larger dataset. In MeInfoText 2.0, the associations were extracted automatically by a text mining system that implements two models, and the reported precision/recall are 94.7% / 90.1% and 91.8% / 90%, respectively for the two models. However, the systems/software that is used in [17,18] to extract the associations and the utilized datasets to train and test the systems were not available during the time of our study. Therefore, we could not perform an independent comparison between the text mining tools used for creating MeInfoText and MeInfoText 2.0 and our system, using our datasets or using data employed in MeInfoText 2.0. Thus, we only report their published results. We want, however, to indicate that performance of our system is assessed based on a much larger manually-classified datasets (we provide to public the annotated PubMed abstracts we used), and we also provide publicly accessible text-mining tool that extracts such information.

Several methods have been developed to extract associations based on similar association structure. Hakenberg et al. [45] developed seven methods to extract twelve different types of associations between different biomedical entities including gene-disease, gene-drug, drug-diseases, mutation-disease, etc. These methods depend on co-occurrence of pairs of named entities and aim to rank the associations based on the confidence. Chun et al. [46] developed a method based on filtering falsely identified named entities to extract associations between genes and diseases. However, methods that are based on co-occurrence of pairs of named entities generate a large number of false positive relations. In our case, the goal of the machine learning method we developed is to determine if a given co-occurrence of named entities constitutes an association. Coulet et al. [47] developed a method based on syntactic parsing to extract associations between pairs of genes, drugs and phenotypes. Unlike other methods that depend on simple co-occurrence, their method depends on analyzing the syntactic structure of sentences to identify the type of associations between named entities such as 'inhibits', 'induces', 'causes', etc., and it is more flexible than rule-based approaches. However, the main drawback of syntactic parsing methods is the low recall, and it requires a large corpus so that there are several opportunities to identify the associations [47].

## Discussion

DTM is used in a traditional approach to represent in a summarized way portions of text (e.g. documents or sentences). In this study, we introduced PWMs as a new method for summarized text representation. To the best of our knowledge, how we used PWMs and the specific scoring of the sentences by them seem to be new in text mining. One advantage of the PWM approach is that it can be applied to sentences that contain multiple patterns. In such cases one can discriminate between classes of patterns. This, on the other hand, is not possible when DTM is used. With DTM each sentence is considered irrespective of the number of patterns the sentence contains. DTM approach does not distinguish between positive and negative patterns contained within the same sentence, and cannot determine the number of positive patterns in the sentence. Therefore, we used DTM approach for sentences with single pattern only to determine if the patter is positive or not. 

It should be noted that in most cases DTM approach generates a large number of features and thus may require a features selection step (i.e., keywords selection). However, PWMs approach does not produce a large number of features. In our study it generated only 12 features. 

Both approaches show decrease in performance when applied to sentences with one pattern. A possible reason may be that these sentences are shorter than sentences that contain multiple patterns. Sentences with one pattern contain smaller number of words resulting in overall poorer information. This makes the classification task more challenging. Also, when the PWM approach was applied to the entire set C, it outperformed the DTM approach in the cases when the random forest, C4.5 and random tree classifiers are used. This shows that the scores that were generated by PWMs can capture the characteristics of each class of sentences better than the DTM approach. However, when the DTM approach was applied to set C on sentences with one pattern only, it outperformed the PWMs approach.

The previous analysis helped us understand the strengths and weaknesses of each approach, and the conditions in which each approach performed the best. PWMs approach performed the best when applied on sentences that include several patterns. Also, DTM approach performed better than PWMs approach when applied on sentences that include only one pattern. Therefore, there was a need to implement a hybrid approach, which could capture the strengths of PWM and DTM approaches, and possibly reduce their weaknesses. When we applied the hybrid approach on set C with random forest, C4.5, and random tree algorithms, it performed better than either of the PWMs and DTM approaches.

## Conclusion

In essence, development of a new way for summarized text representation using PWMs and the scoring mechanism is one of the main contributions we made in this study. One of the great advantages of the PWM approach is that it generates relatively small number of features and works well with sentences that contain more than one pattern. The PWMs approach described in this study can be easily generalized. 

Another major contribution of our study is development of a method for extracting associations between methylated genes in diseases as a combination of PWM and DTM approaches, as well as development of a system, DEMGD, for automated extraction of these associations. This system is the first one publicly available for this purpose. Our methodology and the DEMDG system have been developed on and tested using manually curated data. Both work with any free text.

Moreover, our results were derived based on a comparative study we have conducted between twelve algorithms using the three approaches (PWMs, DTM, and Hybrid). Such comparison study can help in selecting the most suitable method for specific association extraction problem.

We plan to use DEMGD to analyze the whole PubMed database to extract all associations between methylated genes and diseases. We believe that our system, being publicly available, will provide good service to the research community in this field. 

## Supporting Information

Information S1 **Description of different computations.**
(PDF)

Table S1 **Details on applied parameters.** This table explains the kind and range of parameters which were tested when we applied the machine learning algorithms.
(DOC)
