# Supplementary material for: Combining Position Weight Matrices and Document-Term Matrix for Efficient Extraction of Associations of Methylated Genes and Diseases from Free Text
Source: PLoS One. 2013 Oct 16;8(10):e77848. doi: 10.1371/journal.pone.0077848 (PMC3797705; doi:10.1371/journal.pone.0077848)
Supplement: Information S1 — Description of different computations. (PDF) [file pone.0077848.s001.pdf]

Combining position weight matrices and document-term matrix for efficient extraction of associations of methylated genes and diseases from free text

Arwa Bin Raies[1], Hicham Mansour[2], Roberto Incitti[2] and Vladimir B. Bajic[1,*]

[1]Computational Bioscience Research Centre (CBRC), Computer, Electrical and Mathematical Sciences and Engineering Division (CEMSE), King Abdullah University of Science and Technology (KAUST), Thuwal, 23955-6900, Saudi Arabia

[2]Bioscience Core Laboratories, King Abdullah University of Science and Technology (KAUST), Thuwal, 23955-6900, Saudi Arabia

Supplementary Information S1

Description of different computations

Information Gain

$$G(t) = -\sum_{i=1}^{m} P(c_i)\log P(c_i) + P(t)\sum_{i=1}^{m} P(c_i|t)\log P(c_i|t) + P(\bar{t})\sum_{i=1}^{m} P(c_i|\bar{t})\log P(c_i|\bar{t})$$

where m is the number of classes, $P(c_i)$ is the probability of the class c_i, $P(t)$ is the probability of the term t, $P(c_i|t)$ is the probability of a class given the term, $P(\bar{t})$ is the probability that the term does not appear, and $P(c_i|\bar{t})$ is the probability of the class given the term does not appear.

Term-Frequency Inverse-Document-Frequency

$$TF - IDF = tf \times log\frac{N}{n}$$

where tf number of times a term occurs in a sentence, N is the total number of sentences, and n is the number of sentences in which the term appears.

Z-score Normalization

$$\grave{v} = \frac{v - \bar{A}}{\sigma_A}$$

where v is a value of attribute A, $\bar{A}$ is the mean of the attribute A, and σ_A is the standard deviation of the attribute A.
