# Supplementary material for: Combining Position Weight Matrices and Document-Term Matrix for Efficient Extraction of Associations of Methylated Genes and Diseases from Free Text
Source: PLoS One. 2013 Oct 16;8(10):e77848. doi: 10.1371/journal.pone.0077848 (PMC3797705; doi:10.1371/journal.pone.0077848)
Supplement: Table S1 — Details on applied parameters. This table explains the kind and range of parameters which were tested when we applied the machine learning algorithms. (DOC) [file pone.0077848.s002.doc]

Table S1. Details on applied parameters. This table explains the kind and range of parameters which were tested when we applied the machine learning algorithms. For example, we applied random tree algorithm with the PWM approach using 2, 4, 6, 8, 10 and 12 randomly selected features.

| **Algorithm** | **Parameter** | **values** |
| --- | --- | --- |
| **Random Forest** | Number of trees | 5, 10, 15, 20, 25, 30 |
|  | Number of randomly selected features | For PWMs approach {2, 4, 6, 8, 10, 12}  For DTFM approach {5, 10, 15, 20, 25, 30} |
| **SVM** | Cost, Coef0, Degree | 2^n^ , n = {0,1, 2, 3, 4, 5} |
|  | Gamma | 1^/2n^ , n = {0,1, 2, 3, 4, 5} |
| **KNN** | Number of nearest neighbours | 2^n^ -1, n = {1, 2, 3, 4, 5} |
| **C4.5** | Confidence | 0.1, 0.3, 0.5, 0.7, 0.9 |
| **Random Tree** | Number of randomly selected features | For PWMs approach {2, 4, 6, 8, 10, 12}  For DTFM approach {5, 10, 15, 20, 25, 30} |
